# Fetal Wellbeing Monitoring: A Review Article

**DOI:** 10.7759/cureus.29039

**Published:** 2022-09-11

**Authors:** Suhani Jain, Neema Acharya

**Affiliations:** 1 Department of Obstetrics and Gynaecology, Jawaharlal Nehru Medical College, Datta Meghe Institute of Medical Science (Deemed to be University), Wardha, IND

**Keywords:** genetic coding, cell-free fetal d.n.a, doppler, pregnancy, antenatal, fetal assessment

## Abstract

While assessing maternal health is relatively easy, assessing fetal well-being has always been tricky. This has led to tremendous technological development in fetal well-being assessment, thus bridging the gap between biotechnology and antenatal medicine. It is broadly divided into early pregnancy, late pregnancy, and during labour assessment. While the early assessment involves genetic check-ups and malformations, the late pregnancy check-ups aim at delivering a healthy fetus at term by normal vaginal delivery. The early tests can be invasive or non-invasive. Non-invasive include cell-free fetal DNA assessment and fetal cell-based assessment. Invasive tests include amniocentesis and chorionic villous sampling. These are followed by chromosomal microarray and next-generation sequencing. Under this procedure, exome sequencing is done, which is either clinical or whole. Sequencing of the whole genome can also be done. A recent advancement is pre-implantation genetic testing. These are mainly useful in identifying monogenic disorders for which the locus causing disease is identified beyond any doubt. In late pregnancy, the most commonly used test is biophysical. It works on the principle that an increase in the fetal heart rate occurs in conjugation with fetal movements. The next widely employed technology is Doppler, which is used to know fetal heart rates, valve timing intervals, and umbilical artery waveforms. Cardiotocography is also widely used both during pregnancy and during labour. It measures the fetal heart rate while correlating it with uterine contractions. Wireless fetal and maternal heart monitoring and telemonitoring are recent upcoming fields.

## Introduction and background

Pregnancy and the birth of a healthy infant are among the most critical events in the life cycle. The whole of obstetrical science is dedicated to making this event as uneventful as possible, resulting in a healthy baby and mother. The most significant hindrance to this goal is that majority of the fetal deaths occur in utero. The top causes of fetal death are chronic hypoxia leading to intrauterine growth retardation, maternal complications, congenital fetal malformations, and chromosomal abnormalities. While maternal factors can be easily detected and managed, fetal complications require a higher level of diagnostic and management capabilities. One of the significant difficulties obstetricians face is the low accuracy of clinical methods in diagnosing fetal well-being. As necessity is the mother of invention, technological achievements in antenatal fetal assessment have reduced fetal mortality and improved the standard of living. Technology has evolved from invasive chorionic villous sampling to non-invasive cell-free fetal DNA detection [1]. These non-invasive techniques save considerable time, which is then used to prevent complications [2,3]. 

While technological advancements are being made exponentially, the rationality of these advancements must be checked. The checkpoints for antenatal fetal tests are, firstly, that the test must provide information superior to clinical examination. As most of the world struggles with primary health care, costly tests with no substantial benefit are a financial burden on the already underprivileged ones. Secondly, the test results should help manage fetal health and improve perinatal outcomes. Giving a dire prognosis with no management plan adds nothing to the growth of medical science and patient well-being. Thirdly, the advantages of tests must be superior to the potential risks.

The most significant benefit of an apt fetal well-being assessment test is that if the fetus is found compromised, the measures that can be taken to combat the issues are more than often basic. They include bed rest for the mother, follow-up fetal surveillance, drug therapy, urgent delivery, neonatal intensive care, and, in unfortunate cases, abortion. The bridging of perinatal medicine and biotechnological sciences is thus the need of the hour.

## Review

Assessment of fetal well-being can be done in three phases of pregnancy: early pregnancy, late pregnancy, and during labour.

Early pregnancy

Prenatal Genetic Testing

Fetal assessment in early pregnancy mainly detects chromosomal and congenital abnormalities. Prenatal genetic screening has evolved from age-old invasive techniques like chorionic villous sampling to non-invasive techniques like blood sampling or cell-free fetal DNA [1]. Non-invasive techniques - The current approach of using cell-free fetal DNA provides easier, less labour-intensive, and less time-consuming ways to work with fetal DNA [2,3]. But the cell-free fetal DNA technique comes with some drawbacks which have to be kept in mind while implying this technique. According to Haghiac et al., maternal cell-free DNA levels are affected by certain factors. These are increased in obese pregnant women [4]. Although the cell-free fetal DNA levels are unaffected by the weight of the mother, the ratio of fetal fraction decreases with increased maternal fraction. Thus, maternal obesity negatively impacts the diagnostic capabilities of this test [1].

Another recent development in non-invasive prenatal testing is the fetal cell-based approach. This was developed to overcome the drawbacks of the assessment of cell-free fetal DNA. Fetal cells used in this procedure are fetal nucleated red blood cells and trophoblasts. Trophoblasts are preferred over nucleated fetal red blood cells as the red blood cells are present in low concentrations and have markers with low specificity. One of the significant drawbacks of the fetal cell-based approach is fetoplacental mosaicism which is overcome by a silicon-based nanostructured microfluidic platform. It captures circulating fetal neonatal red blood cells and extravillous cytotrophoblasts [5].

Invasive techniques for prenatal genetic testing are employed as confirmatory tests after abnormalities are detected in the non-invasive techniques. These are not used as screening tests as they threaten abortion. Amniocentesis is popularly done, especially in areas that lag in technological aspects. Amniocentesis is followed by karyotyping, which detects the abnormality. One of the advances in invasive testing is performing chromosomal microarray after amniocentesis instead of karyotyping. They are capable of detecting clinically relevant sub-microscopic copy number variants [6,7]. Instead of the above-mentioned procedures, next-generation sequencing can also be done after amniocentesis or chorionic villous sampling. Under this procedure, exome sequencing is done, which is either clinical or whole. Sequencing of the whole genome can also be done.

Another approach for determining possible genetic defects in a fetus is by performing pre-implantation genetic testing. It is done to diagnose any genetic disorder caused by variation in a single gene, for which the locus causing disease is identified beyond any doubt [8,9]. The loci included are nuclear (X-linked, autosomal, dominantly or recessively inherited) or mitochondrial (maternally inherited) and involve (likely) pathogenic genetic variant(s) [10]. It is also used for detecting chromosomal structural defects and aneuploidy [11]. A biopsy is done at the blastocyst stage, after which a genome-wide technology is used to detect abnormalities. Genotype, as well as chromosome copy number, is obtained after the test result [8]. One of the most dreaded drawbacks of this is its misuse in eugenics, which is the main reason why this procedure has not received a legislative and ethical nod in many parts of the world.

Thus, as genetic testing methods are rapidly evolving along with expanding bioinformatics capacities, the scenario in prenatal genetic medicine is changing continuously.

Late pregnancy

The main objective of conducting fetal assessment in late pregnancy is to prevent fetal death and to avoid unnecessary interventions during labour. Methods involved while performing fetal assessment can be clinical, biochemical, and biophysical.

Biochemical Tests

These mainly assess fetal lung maturity and placental function assessment. Though this method gained popularity at the time of its introduction in the 1960s, multiple randomized controlled trials done after that show that they hold almost little to no diagnostic and prognostic value due to low sensitivity and specificity [12].

Biophysical Tests

These are performed to check for uteroplacental insufficiency since fetal hypoxia is one of the leading causes of fetal mortality. It can be started from 32 weeks of gestation and continued till the term. It works on the principle that an acceleration in a fetal heartbeat is observed when fetal movements occur. In other words, it takes advantage of the coordination between fetal neurological status and the cardiac system [13]. They are therefore more helpful in identifying fetal well-being rather than fetal distress and thus are generally used as just screening tests. A wide variety of tests are used under this. They include a count of fetal movement, ultrasonography, cardiotocography, non-stress test, fetal biophysical profile, Doppler ultrasound, vibroacoustic stimulation test, contraction stress test, and amniotic fluid volume.

Fetal movement count - Fetal movements in the womb correspond to the fetal health inside. It helps in alerting the mother and caregiver regarding any fetal distress. There are several methods by which the mother can count fetal movements. The most popularly used two methods are Cardiff count ten formula and daily fetal movement count [14,15]. In the Cardiff formula, the patient starts counting from 9 am and counts till there are ten counts. Normally these ten movements should occur within 12 hours. In the daily fetal movement count method, three counts each of one-hour duration are recommended. The total count is multiplied by four, and a count of less than 10 indicates fetal compromise. Women are a lot more compliant with the daily fetal movement count method. A decrease in fetal movement signals fetal damage [16]. It can lead to preterm labour, intrauterine growth retardation, stillbirth, and emergency caesarean section [17]. A disadvantage of fetal movement counting is that it may cause unnecessary anxiety to the mother as fetal movement can be reduced when the fetus is sleeping, in obesity, and with an anterior placenta.

The non-stress test is used from 32 weeks of gestation. The principle behind this test is that the fetal heart rate fluctuates with fetal movements, which is measured through electrical monitors. It helps determine intrauterine death and other neonatal complications [13]. It has high false-negative results [18]. Vibroacoustic stimulation - It helps in decreasing the false-negative result due to the non-stress test [18,19]. One of the causes of the false-negative result is fetal sleep. Vibroacoustic stimulation helps in changing the non-rapid eye movement sleep of the fetus to rapid eye movement sleep, thus changing from a quiet to an active state.

Modified Biophysical profile scoring - It is observed for 30 minutes. The normal score is two, and the abnormal is zero. The parameters used for biophysical scoring are the non-stress test, fetal breathing movements, gross body movements of the fetus, fetal muscle tone, and amniotic fluid pocket assessment. Each of the following criteria is given a score of two (Table 1).

**Table 1:** Biophysical profile scoring.

	NORMAL	SCORING
Non-stress test	Reactive	2
Fetal breathing movements	More than/equal to one episode lasting more than 30 seconds	2
Gross body movements	More than/equal to three movements	2
Fetal muscle tone	More than/ equal to one episode of active extension and flexion	2
Amniotic fluid	A vertical pocket of more than/equal to 2cm.	2

Doppler Ultrasound

Fetal heart rates and valve timing intervals are one of the most common methods employed to monitor fetal heart rate in the last trimester, which is done using the Doppler ultrasound. Though, the specificity of this test is low. A new method of measuring fetal cardiac interval from Doppler ultrasound has been developed. This combines empirical mode decomposition and hybrid support vector machines-hidden Markov models [20]. Another method presented combined Doppler and ECG. The beat-to-beat fetal heart rate received from the Doppler was compared with the abdominal lead of fetal ECG [21]. Doppler velocimetry of the middle cerebral artery (MCA) is a technique for determining flow impedance/resistance in fetal brain circulation. Vasodilation of the MCA is thought to be a compensatory mechanism known as the "brain sparing effect" [22]. It works on the principle that in fetal hypoxia, the body, to save the vital organs, redirects the blood towards them, thus causing vasodilatation. Although fetal Doppler and electrical signaling techniques are widely used in obstetrics, they have their own sets of drawbacks. Their specificity is very low, and thus the rates of false-positive results are high. From the signals from the abdomen, non-stationary signals are obtained.

Cardiotocography 

**Figure 1:**
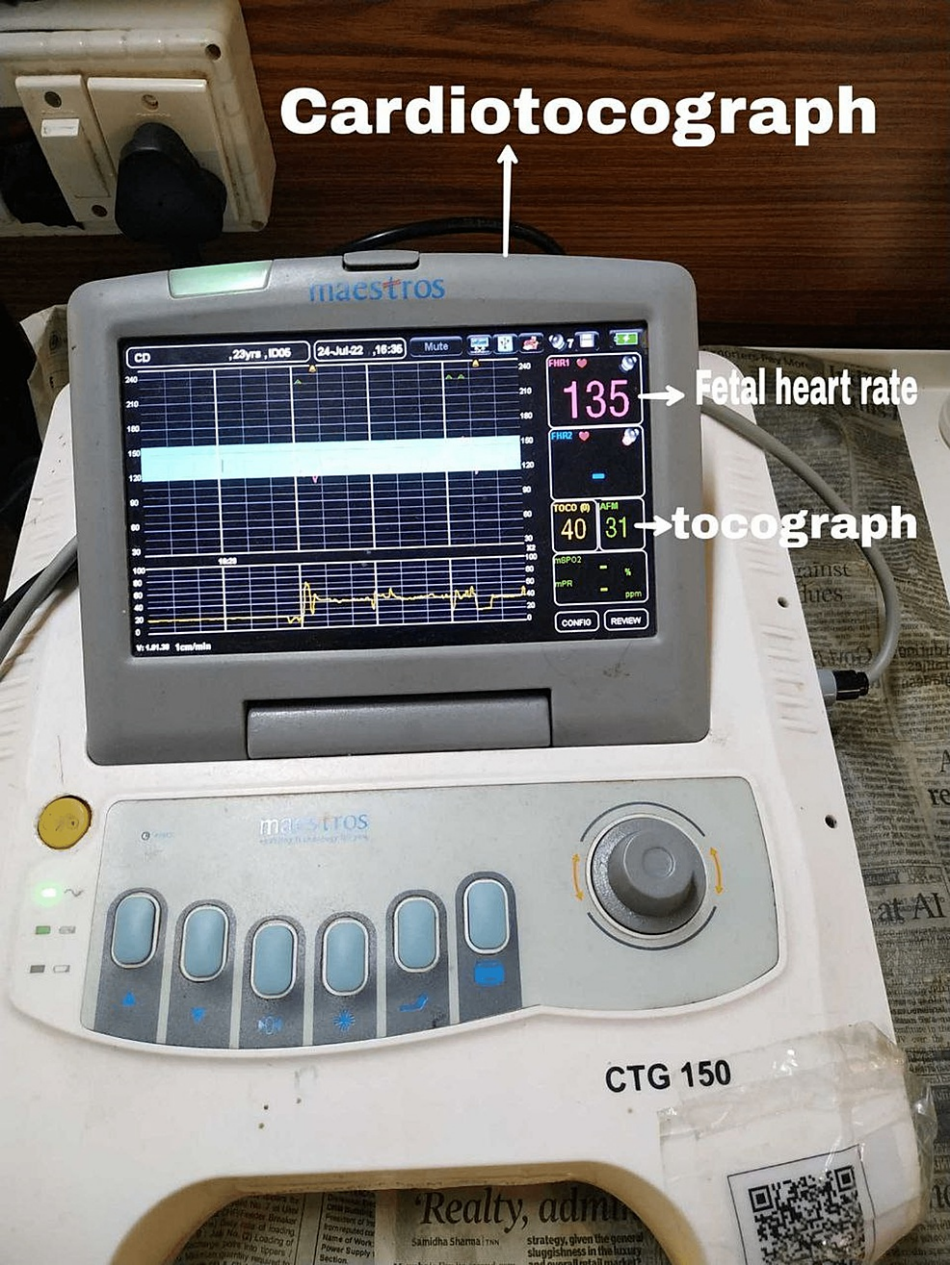
Cardiotocograph.

It is an electronic method of assessing fetal heart rate using ultrasound transducers (Figure 1). One transducer is placed on the abdomen of the mother for fetal heart rate, and the other is placed over the fundus of the gravid uterus to record uterine contractions. This makes cardiotocography useful not only during the antepartum period but also during labour [23]. A strip of paper is used to simultaneously record the fetal heart rate and uterine activity. The aspects of the fetal heart rate which are measured are its baseline rate, baseline variability, accelerations, and decelerations. The link between fetal heart rate and uterine contraction time is also investigated. It can be used in isolation as in a non-stress test or in conjunction with a contraction stress test in which the response of fetal heart rate to stimulated uterine contraction is seen [23]. It is usually done in the third trimester after 28 weeks of gestation [24]. Indications for cardiotocography and gestational age determine the frequency of testing, and thus it varies from patient to patient. It usually ranges from weekly to three times a day.

While the fetal heart rate and tocograph aspect of cardiotocography are very well utilised throughout the world in felt assessment, the autograph trace is not utilised to its full potential. The clinical value of maternal-fetal movement count is very less as the quantification of fetal movement is very subjective. The autograph helps in the quantification of these fetal movements and thus helps in resolving the discrepancies. The high-frequency signal is distinguished and recorded as fetal cardiac activity, whereas the low-frequency data is interpreted as fetal movement. The autograph measures fetal movements and correlates them with fetal size in relation to gestation and fetoplacental Doppler parameters [25].

Umbilical Artery Waveform 

Pulsed Doppler (two MHz) ultrasound from the umbilical artery and real-time B mode are combined to obtain blood velocity waveforms [26].

Wireless Fetal and Maternal Heart Monitoring 

The economic imbalance within the society, non-proportional doctor-to-patient ratio, inaccessibility to primary healthcare, and requirement of multiple health centre visits during pregnancy lead to decreased visits of patients to medical health care providers, which in turn increases fetal and maternal mortality. These, combined with the aforementioned drawbacks of present fetal assessment techniques like Doppler, necessitate the inclusion of wireless fetal and maternal heart monitoring in areas where healthcare services are backward. The drawbacks of present-day fetal well-being assessment techniques are that in cardiotocography, utility in high body mass index females is very low, Doppler deposits energy particles in tissue, and they lack automated analysis [27]. The monitoring device must fulfill the following requirements; it should have easy self-applicability without the need for device repositioning by a professional, it should distinguish between fetal heart rate and maternal heart rate, it should provide continuous monitoring of fetal heart rate, and have low false-positive rate and last but not the least it should be comfortable and cheap [28].

All the aforementioned challenges are overcome by Invu, a fully remote, medical-grade maternal-fetal monitoring solution. It is based on fetal and maternal echocardiography data. 

Telemonitoring

In this era where the whole world is available to us at the tips of our fingers, it makes sense to intersect telecommunication and prenatal medicine. With about a million healthcare apps and almost everyone owning a smartphone, the potential for growth in telemedicine is tremendous. It is mainly used in high-risk pregnancies, especially those at risk of preterm labour [28,29].

During labour

Cardiotocography

It helps in assessing fetal hypoxia and thus plays a major role in determining if the delivery should be instrumental vaginal or caesarean section [30].

Blood Sampling of the Fetal Skull

The most often utilised method is the fetal scalp blood sample, which allows for direct detection of acidosis biochemical markers such as pH, base deficit, and lactate. The sampling procedure needs the cervix to be dilated a minimum of 3 cm for sufficient visibility of the fetal scalp. The scalp is visualised using an amnioscope which is inserted vaginally. A tiny incision is performed on the fetal scalp and about 30 to 50 microlitre of blood into a test tube with heparin which prevents coagulation. A blood gas analyser is then used to test the sample [31,32].

Because of its capacity to discriminate between different forms of acidosis, lactate in blood monitoring has been highlighted as a viable option for intrapartum fetal monitoring [31,32]. Fetal scalp lactate during labour and umbilical artery lactate at delivery has been demonstrated to be more accurate than pH in predicting a poor newborn state and hypoxic-ischemic encephalopathy [33,34].

## Conclusions

During early pregnancy, non-invasive fetal well-being assessment comprises a cell-free fetal DNA approach and a fetal cell-based approach. Cell-free fetal DNA is affected by maternal body fat, while this drawback is not present in the fetal cell-based approach. The fetoplacental mosaicism is the disadvantage of the fetal cell-based approach. The invasive techniques involved are mainly used as diagnostic tools and not screening as they can lead to abortion. Amniocentesis followed by karyotyping or chromosomal microarray, or next-generation sequencing, is performed to look for genetic defects in the developing fetus. Exome or whole genome sequencing can also be done. Pre-implantation genetic testing and biopsy at the blastocyst stage are advanced methods for detecting aneuploidy and chromosomal structural defect.

In late pregnancy, biochemical tests for feta maturity are performed, but their diagnostic value is questionable. Biophysical tests for fetoplacental insufficiency are widely performed. Fetal movement counted by the mother, though it is fairly easy, it has little value clinically as it is subjective. Non-stress tests with or without vibroacoustic stimulation correlate fetal heart rate with fetal movements. Doppler ultrasound has wide usage. It is used in fetal heart rate monitoring, middle cerebral artery velocimetry, and valve timing interval. Cardiotocography is a test that can be used both antenatally and intranasally. It comprises of fetal heart rate aspect, tocograph aspect, and the less commonly used autograph. Wireless fetal and maternal monitoring and telemonitoring are emerging domains of technology. During labour, cardiotocography and fetal skull blood sampling are widely used.
